# Comparison of Intravenous Anti-hypertensives for Preoperative Blood Pressure Control in Hypertensive Disorders of Pregnancy and Effect of Oral Labetalol

**DOI:** 10.7759/cureus.32858

**Published:** 2022-12-23

**Authors:** Ranju Singh, Jyotika Kumar, Aruna Jain, Manju Puri

**Affiliations:** 1 Anaesthesiology, Lady Hardinge Medical College, Delhi, IND; 2 Obstetrics and Gynaecology, Lady Hardinge Medical College, Delhi, IND

**Keywords:** hypertension, pregnancy, pre-eclampsia, hydralazine, labetalol

## Abstract

Background and aims: Intravenous hydralazine and labetalol are recommended as first-line anti-hypertensives for controlling severe hypertension in pregnancy. Our study aimed at identifying the most effective drug with minimum side effects for preoperative management of severe hypertension in parturients scheduled for Caesarean delivery (CD). We also studied the effect of these drugs on patients already on oral labetalol in the antenatal period.

Methods: A prospective observational study was done on 162 hypertensive parturients scheduled to undergo emergency CD who received hydralazine or labetalol in the preoperative period. Demographic data, booking status, hemodynamic data, time taken to reach adequate control of blood pressure (BP), drug efficacy, the incidence of persistent hypertension, adverse effects associated with the drugs, and maternal and fetal outcomes were noted.

Results: The time taken for the control of BP was similar with both drugs (p-value = 0.425). The mean number of doses required to achieve target BP was significantly less with hydralazine compared to labetalol (p-value = 0.009). Patients on tablet labetalol in the antenatal period were poorly controlled when put on the same drug intravenously but had better control with hydralazine (p-value = 0.005). The incidence of persistent hypertension was lower in patients treated with hydralazine compared with labetalol (p-value = 0.008).

Conclusion: Both drugs took a similar time for BP control. However, hydralazine was more efficacious, produced adequate control of BP in a higher number of patients, and had a lower incidence of persistent hypertension.

## Introduction

Hypertensive disorders of pregnancy are one of the most common medical complications of pregnancy, being a major cause of maternal, fetal, and neonatal morbidity and mortality [1]. The ultimate cure for pre-eclampsia and eclampsia is the delivery of the baby. However, maternal and perinatal deaths are significantly reduced with appropriate treatment for the increased blood pressure (BP) [2], and there is enough evidence available in the literature to indicate that the mismanagement of hypertension in pregnancy before CD can lead to serious maternal and fetal complications [3].

Although intravenous (IV) labetalol and hydralazine have been recommended as first-line medications, the treatment of hypertensive crises associated with pre-eclampsia and eclampsia remains under investigation. Despite the many studies and meta-analyses on the subject, there is no definitive consensus or recommendation regarding the best anti-hypertensive for controlling a hypertensive crisis preoperatively [4]. A Cochrane systematic review concluded that there was no evidence that one anti-hypertensive agent was preferable to the others for improving outcomes for women with SBP greater than 170 mmHg and DBP greater than 110 mmHg during pregnancy and their babies [5]. Anti-hypertensive management of patients at our institution, which is a referral center for maternal and child health, prior to an emergency CD depends on the choice of the treating obstetrician and the availability of the drug, with no specific clinical protocol. The study aims at highlighting the drug that achieves faster BP control, the efficacy of drugs, the drug's effectiveness in patients already receiving oral anti-hypertensive medications during their antenatal period, and comparing the side effect profile.

## Materials and methods

This was a prospective observational study conducted in the Department of Anaesthesiology of a tertiary care hospital from November 2016 to March 2018, after approval by the Institutional Ethical Committee. The study was registered at the Clinical Trials Registry India (CTRI/2018/05/013709). We studied 167 pregnant patients with hypertensive crises scheduled for emergency CD under spinal or general anesthesia. After a detailed pre-anesthetic check-up and a written informed consent, all hypertensive pregnant patients aged 21-45 years with single or multiple pregnancies who had a BP ≥ 160/110 with a gestational age >24 weeks and were given anti-hypertensive agents intravenously prior to the administration of anesthesia were included in the study.

Demographic data, including age, weight, parity, and gestational age, were noted. The patient's attendance at the antenatal clinic (booked/unbooked) and any anti-hypertensives that were started in the antenatal period were noted. The blood pressure at which the patient presented for labor and delivery and the blood pressure at which the patient was taken for CD were noted. Anti-hypertensive drugs administered included hydralazine and labetalol as per the availability and choice of the attending anesthesiologist in the pre-operative room. Reconstitution of IV hydralazine was done by dissolving hydralazine 20 mg powder in 2 ml of sodium chloride 0.9% in the vial, then it was further diluted with 18 ml of sodium chloride 0.9% to give a final concentration of 1 mg/ml. Labetalol did not require reconstitution as it is available in ampoules of 4 ml, each milliliter containing 5 mg. Hydralazine (5 mg) was given IV over two minutes. After 20 minutes, BP was measured, and if SBP persisted ≥160 mmHg or/and if DBP ≥110 mmHg, 10 mg IV was repeated. The BP was measured again after 20 minutes. If BP was still above the desired limit, another 10 mg was administered, and if after 20 minutes BP was still uncontrolled, then the drug was changed to labetalol. Labetalol 20 mg was given IV initially over two minutes. After 10 minutes, the BP was measured, and if SBP persisted ≥160 mmHg or/and if DBP ≥110 mmHg, the dose was doubled (40 mg). If the BP was not controlled with this second IV dose, the next dose of 80 mg was given over two minutes. This 80-mg dose was repeated if adequate control of BP was not achieved (maximum total dose of 300 mg). If the BP was still not controlled, then the drug was changed to hydralazine. Magnesium sulfate was given to parturients in both groups for seizure prophylaxis.

The primary objective was the time taken to reach adequate control of BP (SBP 140-150 mmHg and/or DBP 90-100 mmHg) with intravenously used anti-hypertensive drugs (labetalol or hydralazine) in patients with hypertensive disorders of pregnancy undergoing CD. The secondary objectives were to study the efficacy (number of doses required for adequate control), the number of patients who achieved adequate control of BP with each drug, the rate of persistent hypertension (requirement of changing anti-hypertensive medications), adverse effects, and the maternal and fetal outcomes with both drugs.

These patients were monitored for the control of BP (every 10 minutes) till they were taken up for CD. The choice of anesthesia and intraoperative BP were noted. Postoperatively, the patient was followed six hours a day for 24 hours, and a note was made of persistent hypertension and the drugs administered for the same.

The estimated sample size was based on the primary objective of the time taken to achieve the target BP. We defined a relevant difference of 10 minutes in the mean time between the two groups. Thus, with the sample size of at least 63 patients per group, there was 80% power with an effective size of 0.50 at an alpha of 0.05 to detect a difference of 0.05 between the two groups. Assuming a 20% loss in sample processing, at least 75 patients in each group were required.

Statistical analysis was performed by the Statistical Package for Social Sciences program for Windows, version 17.0 (Chicago, Illinois). Normally distributed continuous variables were compared using the unpaired t-test, whereas the Mann-Whitney U test was used for those variables that were not normally distributed. Categorical variables were analyzed using either the chi-square test or Fisher’s exact test. A p-value < 0.05 was considered statistically significant.

## Results

Out of the 167 patients enrolled, five patients underwent emergency CD in view of acute and severe fetal distress (fetal bradycardia, tachycardia, repetitive variable decelerations, and late decelerations) without waiting for adequate control of BP and were excluded from the study. Therefore, a total of 162 patients were included, of whom 85 received labetalol (group L) and 77 received hydralazine (group H). The demographic data (parity, gestational age, number of patients booked in the antenatal clinic (ANC), and initial blood pressure) were comparable between the two groups (Table 1). Spinal anesthesia was preferred for CD in the majority of patients in both groups (Table 1). Booked patients were 92; out of these, 39 patients were receiving the tablet labetalol for control of BP in the ANC. Therefore, out of 77 patients who received injection hydralazine, 17 patients were already taking tablet labetalol. Of the 85 patients who received injection labetalol, 22 were on tablet labetalol (p-value = 0.374).

**Table 1:** Patient demographics, booking status and type of anesthesia [parameters expressed as mean with standard deviation (SD)] SBP: systolic blood pressure, DBP: diastolic blood pressure, *H: hydralazine group, ^†^L: labetalol group, ^‡^n: number of patients in each group

Patient parameters	Drug given		p-value
	H* (n=77)	L^†^ (n^‡^=85)	
	Mean ± SD	Mean ± SD	
Age (years)	27.08 ± 3.55	26.76 ± 3.26	0.559
Weight (kg)	58.29 ± 8.18	59.84 ± 7.70	0.750
Nulliparous (frequency)	49 (63.6%)	55 (64.7%)	0.848
Multiparous (frequency)	28 (36.4%)	30 (35.3%)	0.848
Gestational age <37 weeks	27 (35.0%)	22 (25.9%)	0.157
Gestational age ≥37 weeks	50 (65.0%)	63 (74.1%)	0.317
Booked (number of patients)	41 (53.2%)	51 (60%)	0.386
Unbooked (number of patients)	36 (46.8%)	34 (40.0%)	
SBP on arrival (mmHg)	189.53 ± 12.62	191.62 ± 15.46	0.350
DBP on arrival (mmHg)	101.51 ± 9.84	100.92 ± 11.40	0.727
Patients given spinal anesthesia	64 (83.1%)	70 (82.3%)	0.898

The time taken for the control of BP with hydralazine and labetalol was comparable, but hydralazine was more efficacious than labetalol in controlling BP (Table 2). Six patients (7.8%) in group H and 20 (23.5%) patients in group L did not achieve adequate control of BP (p-value = 0.006, Table 3). They were then switched over to labetalol and hydralazine, respectively.

**Table 2:** Time taken and efficacy of each drug for control of blood pressure (parameters expressed as mean with standard deviation) *H: hydralazine group, ^†^L: labetalol group, ^‡^n: number of patients in each group

	Drug given		p-value
	H* (n=77)	L^†^ (n=85)	
Time taken for control of BP in mins (mean ± standard deviation)	21.99 ± 11.62	20.63 ± 10.00	0.425
Number of doses	Number of patients (%)		
1	33 (42.9%)	8 (9.4%)	<0.001
2	31 (40.3%)	29 (34.1%)	
3	13 (16.9%)	24 (28.2%)	
4	0	4 (4.8%)	
5	0	20 (23.5%)	
Mean number of doses	1.74 ± 0.73	2.98 ± 1.3	

**Table 3:** Incidence of persistent hypertension and need for a second anti-hypertensive drug *H: hydralazine group, ^†^L: labetalol group, ^‡^n: number of patients in each group

Number of patients (%)	Drug given				p-Value
	H* (n=77)		L^†^ (n^‡^=85)		
Controlled	71 (92.2%)		65 (76.5%)		0.006
Not controlled (shifted to alternate drug)	6 (7.8%)		20 (23.5%)		0.006
On oral labetalol	Yes	No	Yes	No	
	2 (33.3%)	4	16 (80%)	4	0.005

After BP control, the BP at which patients were taken for CD was comparable in both groups. The SBP on arrival was 189.53 ± 12.62 mmHg in the hydralazine group and 191.62 ± 15.46 mmHg in the labetalol group (p-value = 0.350). The DBP was 101.51 ± 9.84 mmHg and 100.92 ± 11.40 mmHg in the hydralazine and labetalol groups, respectively (p-value = 0.727). Intraoperative mean SBP recordings in group L were significantly higher than those in group H at 10, 20, and 30 minutes (p-value < 0.05) and were comparable at 40, 50, and 60 minutes. Intraoperative mean DBP recordings were comparable between the two groups at all time points. The requirement for intraoperative anti-hypertensive medications was also comparable in both groups. Nine patients in group H required additional anti-hypertensive (labetalol) and 11 patients in group L required additional anti-hypertensive (hydralazine) for BP control intraoperatively prior to CD, even when they achieved adequate control of BP preoperatively (p-value = 0.809). Postoperative mean SBP and DBP were comparable at 0, 6, 12, 18, and 24 hours. Patients who required postoperative control of BP were significantly more common in group L as compared to group H (p-value = 0.008, Table 4). Maternal side effects were significantly lower in group H compared with group L (Table 5).

**Table 4:** Fetal and maternal outcome (parameters expressed as mean with standard deviation) Apgar: appearance, pulse, grimace, activity, respiration; HELLP: hemolysis, elevated liver enzymes, low platelet count, ICU: intensive care unit, *H: hydralazine group, ^†^L: labetalol group, ^‡^n: number of patients in each group

Fetal parameters	Drug given		p-value
	H* (n=77)	L^†^ (n^‡^=85)	
Prematurity (frequency)	27 (35%)	22 (25.8%)	0.157
Apgar score at 1 min	7.48 ± 1.17	8.07 ± 0.99	0.264
Apgar score at 5 min	8.40 ± 1.23	7.74 ± 1.07	0.199
Birth weight (kilograms)	2.37 ± 0.34	2.66 ± 0.32	0.793
Nursery admissions	25 (32.5%)	18 (26%)	0.141
Maternal outcome	Number of patients		
HELLP	2 (2.5%)	3 (3.52%)	1.000
Oliguria	3 (3.89%)	5 (5.88%)	0.772
Seizures	8 (10.38%)	12 (14.11%)	0.471
Pulmonary edema	0 (0%)	1 (1.76%)	1.000
Death	1 (1.29%)	1 (1.76%)	1.000
ICU admission	11 (14.2%)	14 (16.4%)	0.772
Postoperative hypertension	17 (22%)	26 (30.5%)	0.008

**Table 5:** Maternal and fetal side effects *H: hydralazine group, ^†^L: labetalol group, ^‡^n: number of patients in each group

Side effects	Number of patients in each group		p-Value
	H* (n=77)	L^†^ (n^‡^=85)	
Fetal bradycardia	12 (15.5%)	23 (27.0%)	<0.001
Headache	19 (24.6%)	6 (7.0%)	0.014
Maternal hypotension	4 (5.1%)	7 (8.2%)	0.045
Nausea	10 (12.9%)	6 (7.0%)	0.014

## Discussion

In our study, 61.7% of patients were between 25 and 30 years old, going against the fact that preeclampsia predominantly occurs at an advanced maternal age of >35 years. Younger patients suffering from preeclampsia have also been reported by other authors [6-8]. The majority of patients in both groups were nulliparous, reiterating the fact that preeclampsia is more common in primigravida [6,9]. It has been previously reported that severe preeclampsia was more likely to occur among unbooked patients who may not have had the benefit of early diagnosis and monitoring in the ANC [10]. Findings in our study are contrary to this observation. Perhaps the fact that our hospital caters to women from urban areas may be responsible for this finding, which is consistent with the findings of Nombur et al. [6].

Our study demonstrated no superiority of one drug over the other in achieving faster BP control. Similar times taken for the control of BP with both drugs have also been reported by other authors [6,8]. On the contrary, Mmom et al. reported a longer time to reach a target BP in patients receiving IV hydralazine as compared to labetalol [11]. The reason for this disparity was probably related to racial differences in the study population, as beta-blockers have been found to be less effective in controlling BP in patients of African descent.

Fifty-seven percent of patients in the hydralazine group required more than one dose to achieve target BP, as compared to 90.6% of patients in the labetalol group, making hydralazine more efficacious than labetalol. This can be explained on the basis of the pharmacokinetics and pharmacodynamics of the drug. Contrary to our results, a few other studies have observed that the number of patients controlled with a single dose of hydralazine was almost similar to the number controlled with a single dose of labetalol [6,8,11].

The incidence of persistent hypertension in our study was significantly higher in the labetalol group. Similar findings have been reported by Mmom et al., with the disparity being related to racial differences as beta-blockers are less effective in controlling BP in parturients of African descent [11]. In the meta-analysis conducted by Magee et al., it was established that hydralazine was associated with a trend toward less persistent severe hypertension than labetalol [10]. However, few other authors have observed that the incidence of persistent hypertension was comparable with the use of hydralazine or labetalol [6,7,11].

In our study, out of the 20 patients with persistent hypertension in the labetalol group, 16 patients were receiving oral labetalol in the antenatal period. This highly significant result has important clinical implications. It is evident that patients on oral labetalol in the antenatal period were poorly controlled when put on the same drug intravenously, but had better control with hydralazine preoperatively (as the mechanism of action of both drugs was different). Therefore, we can recommend that if a patient develops a hypertensive crisis and is already on oral labetalol, she should be administered hydralazine intravenously for the preoperative control of BP and not labetalol. Despite an extensive literature search, we were unable to find any published data citing the effect of oral labetalol on the control of preoperative BP with IV anti-hypertensives.

Seven of the 20 patients who required intraoperative anti-hypertensive therapy had received GA, and 13 had received SA. Hence, the requirement for intraoperative anti-hypertensive therapy depends on the disease process itself and not on the technique of anesthesia. After the administration of SA, although there was a sympathetic blockade, an elevated BP was still seen, probably due to the chemical and humoral mediators that circulate in the blood of a hypertensive parturient. We were unable to compare these results with any other published literature.

The incidence of fetal bradycardia was higher in the labetalol group. Fetal bradycardia can be a side effect of the drug itself or can result from other causes, such as fetal distress and meconium staining of liquor, the incidence of which was similar in both groups, hence the bradycardia can be attributed to labetalol. The meta-analysis by Magee et al. [10] reported that labetalol was associated with higher chances of neonatal bradycardia, contrary to the observations by Mmom et al. [11]. Maternal side effects were largely comparable in both groups, as has been reported in previous studies [12]. The increased incidence of headache in the hydralazine group is in keeping with the documented side effects of hydralazine. There was no associated symptomatology (like diplopia or nausea) or abnormal reading of BP at any time in any of these patients complaining of headache, suggesting that the headache was not associated with the worsening of preeclampsia. Few authors have reported a similar incidence of headache with both hydralazine and labetalol [6,9,10,13-15].

Maternal hypotension was significantly higher in the labetalol group, probably a side effect of the drug itself and not due to any other cause such as blood loss or hypovolemia. Also, all patients were preloaded equally with 1 liter of crystalloid, and still more patients in the labetalol group developed hypotension. Intraoperative hypotension was managed with an injection of mephentermine 6 mg given intravenously (maximum dose: 30 mg). Intraoperative fluid management was done according to the fluid deficit, maintenance fluid requirements, and surgical losses. Care was taken not to overload the patient, as hypertensive pregnant patients are known to have leaky capillaries. The incidence of nausea was significantly higher in the hydralazine group. The majority of other studies have quoted an equal incidence of nausea with both hydralazine and labetalol, probably because nausea is a multi-factorial symptom and its occurrence can hardly be attributed to a single cause alone [6,7,9,13,16,17].

The outcomes (comparable in both groups) and complications cannot be attributed to one specific anti-hypertensive but are largely the result of the disease process itself. There is hardly any published data that compare the postoperative maternal outcome or rate of ICU admission after CDs in patients receiving hydralazine or labetalol as anti-hypertensives. Postoperative control of BP was better achieved in patients who received IV hydralazine in the preoperative period as compared to those who received IV labetalol.

Our study has some limitations. It is an observational study, so there is a lack of precision as compared to a randomized control trial, and there is a probability of selection bias. The validity, both external and internal, may be compromised. Blood pressure recordings may be affected by pain due to uterine contractions prior to the administration of spinal anesthesia.

## Conclusions

IV hydralazine is the anti-hypertensive agent of choice for preoperative BP control in patients with hypertensive disorders of pregnancy undergoing CD as it is more efficacious, produces adequate control of BP in a higher number of patients, has a lower incidence of persistent hypertension, maternal hypotension, and fetal bradycardia as compared to IV labetalol, and with its use, the number of patients requiring anti-hypertensive agents in the postoperative period is reduced. Both fetal and maternal outcomes are comparable. Hypertensive pregnant patients who undergo emergency CD in view of fetal bradycardia should not be given intravenous labetalol for the control of BP preoperatively. Patients who were receiving tablet labetalol for the control of BP in the antenatal period should not be given IV labetalol for the control of BP preoperatively when scheduled for an emergency CD. However, more patients need to be studied to corroborate our findings.
